# Developing a Japanese version of the Injustice Experience Questionnaire-chronic and the contribution of perceived injustice to severity of menstrual pain: a web-based cross-sectional study

**DOI:** 10.1186/s13030-019-0158-z

**Published:** 2019-07-22

**Authors:** Keiko Yamada, Tomonori Adachi, Yasuhiko Kubota, Takashi Takeda, Masako Iseki

**Affiliations:** 1 Department of Psychology, McGill University, 2001 McGill College Avenue, Montreal, Quebec H3A 1G1 Canada; 2 Department of Anesthesiology and Pain Medicine, Juntendo University Faculty of Medicine, 2-1-1 Hongo, Bunkyo-ku, Tokyo 113-0033 Japan; 3 Pain Management Clinic, Shiga University of Medical Science Hospital, Seta, Tukinowa-cho, Otsu-shi, Shiga 520-2192 Japan; 4 Osaka Center for Cancer and Cardiovascular Diseases Prevention, 1-6-107 Morinomiya, Jyoto-ku, Osaka 536-0025 Japan; 5 Takashi Takeda; Division of Women’s Health, Research Institute of Traditional Asian Medicine, Kindai University, 377-2 Ohno-higashi, Osaka-sayama, Osaka 589-8511 Japan

**Keywords:** Dysmenorrhea, Perceived injustice, The Injustice Experience Questionnaire

## Abstract

**Background:**

Menstrual pain causes low quality of life among women of reproductive age, and often interferes with daily activities. Perceived injustice is a cognition linked to adverse symptoms. The aims of this study were to develop a Japanese version of the Injustice Experience Questionnaire-chronic (IEQ-chr-J), and to examine if perceived injustice is associated with pain intensity and impairment from menstruation.

**Methods:**

We investigated 130 Japanese women (aged 20–45 years) with menstrual pain in the past 3 months using online self-administered questionnaires. We examined the psychometric properties of the IEQ-chr-J including: structural validity; internal consistency; and test-retest reliability (intra-class correlation coefficients; ICC). Concurrent validity was examined by correlations among the IEQ-chr-J, the Pain Catastrophizing Scale (PCS), the Hospital Anxiety and Depression Scale (HADS), a numerical rating scale (NRS) for maximum/average menstrual pain, and the Brief Pain Inventory (BPI) pain interference domain. We used multivariable regression analysis to investigate the association between perceived injustice and severity of menstrual pain, after excluding 10 hormone drug users.

**Results:**

The IEQ-chr-J showed sufficient validity and reliability (Cronbach’s α = 0.96, ICC 0.75, [95% confidence interval (CI): 0.61–0.88]. Pearson’s correlation coefficients for the IEQ-chr-J, PCS, HADS anxiety, HADS depression, NRS, and BPI pain interference ranged from 0.27–0.65. The IEQ-chr-J was correlated with impairment due to menstrual pain (ICC 0.36, 95% CI: 0.14–0.58), an independent diagnosis of endometriosis, anxiety, and depression, but not with maximum or average pain intensity.

**Conclusions:**

The IEQ-chr-J has acceptable psychometric properties, and perceived injustice is associated with impairment from menstrual pain.

**Electronic supplementary material:**

The online version of this article (10.1186/s13030-019-0158-z) contains supplementary material, which is available to authorized users.

## Background

Menstrual pain leads to low quality of life among women of reproductive age. Severe menstrual pain may also interfere with women’s daily life, including absence from work or school. Previous studies reported that severe dysmenorrhea impaired daily life for 7–15% of women of reproductive age and for 41% of women aged under 26 years [1, 2].

Perceived injustice is a belief linked to adverse psychosocial symptoms such as depression and anxiety, and encompasses blame and unfairness as well as severity and irreparability of loss caused by illness [3]. Perceived injustice is also a unique psychosocial factor associated with chronic pain; for example, pain due to whiplash syndrome, fibromyalgia syndrome, or osteoarthritis [3–6]. However, an association between perceived injustice and menstrual pain has not yet been reported. A previous study showed that injured patients with perceived injustice were resistant to rehabilitation and it was difficult to support them to return to work [3]. For such cases, risk-targeted behavioral interventions designed to improve patients’ perceptions of injustice have been developed [7, 8]. If an association between perceived injustice and menstrual pain is identified, risk-targeted behavioral interventions may be a potential treatment for menstrual pain among women with perceived injustice [7–9].

This study aimed to develop a Japanese version of the Injustice Experience Questionnaire-chronic (IEQ-chr-J) and to examine if perceived injustice as measured by the IEQ-chr-J was associated with pain intensity and impairment due to menstruation.

## Methods

### Study population

We investigated 130 Japanese women with menstrual pain (aged 20–45 years) who had not become pregnant in the past 3 months. The new edition of ICD-11 defined chronic pain as pain that lasts or recurs for longer than 3 months [10]. In addition, a system review of chronic pelvic pain by the World Health Organization included menstrual pain [11]. Therefore, we considered repeated menstrual pain over three menstrual cycles as broad-sense chronic pain. Participating women were randomly selected from among women registered with a web research panel. Respondents completed online self-administered questionnaires. Information collected included demographic characteristics, pain-related items, and psychosocial factors regarding patients with chronic pain. None of the respondents were in menses when they completed the questionnaires. Respondents had to complete each questionnaire before proceeding to the next questionnaire (i.e., there were no missing data). When we examined the association between respondents’ characteristics and severity of menstrual pain, we excluded 10 women with hormone therapy because hormone therapy may affect dysmenorrhea.

### Development of the Japanese version of the IEQ-chr

As shown in the Additional file 1, the original Injustice Experience Questionnaire (IEQ) is a tool used to assess perceived injustice among injured individuals [3]. The IEQ-chronic (IEQ-chr) is a modified version of the instrument for individuals with chronic symptoms; we used the Japanese version of this tool to assess perceived injustice among women with menstrual pain [6]. IEQ-chr items are similar to those in the original IEQ, although the word “injury” was changed to “health status” [6]. For example, “this scale was designed to assess how your injury has affected your life” (IEQ) became “this scale was designed to assess how your health status has affected your life” (IEQ-chr) [3, 6]. Similar modifications were made in the Japanese version.

First, we confirmed the validity and reliability of the IEQ-chr-J, as the Japanese version has not previously been validated. We used confirmatory factor analysis (CFA) to evaluate structural validity. Variation and covariation among the 12 items were assessed using fit indices for a one-factor structure model, based on ≥9 points of modification indices produced by the statistical package. We calculated several fit indices: chi square (χ^2^), chi square divided by degree of freedom (χ^2^/df), the root mean square error of approximation (RMSEA) with 90% confidence intervals (CI), the standardized root mean square residual (SRMR), comparative fit index (CFI), and Tucker-Lewis index (TLI). A RMSEA value < 0.08 suggests a good fit, 0.08–0.10 a moderate fit, and > 0.10 a poor fit [12]. SRMR values < 0.09 indicate a good fit [12], and CFI and TLI values close to 0.95 are considered to indicate a relatively good fit [13].

The concurrent validity of the IEQ-chr-J was calculated using Pearson’s correlation coefficients for the Pain Catastrophizing Scale (PCS), Hospital Anxiety and Depression Scale (HADS) anxiety subscale, HADS depression subscale, a numerical rating scale (NRS) for maximum/average menstrual pain, the Brief Pain Inventory (BPI) pain interference domain, and the IEQ-chr-J. The English version of IEQ-chr was significantly correlated with catastrophizing, depression, pain intensity, and pain disability [6]. Therefore, we used PCS, HADS, NRS, and BPI pain interference domain to evaluate concurrent validity. The PCS comprises 13 items on Likert-type scales from 0 to 4, which are converted into 0–52 points to measure exaggerated pain [14]. The PCS was previously used to examine the concurrent validity of the IEQ and IEQ-chr, and was highly correlated with IEQ, IEQ-J, and IEQ-chr scores [6, 14, 15]. Each HADS subscale includes 7 items with responses on a 4-point Likert scale from 0 to 3, which convert into scores up to 21 for anxiety/depressive symptoms in the past week [16].

The internal consistency of the IEQ-chr-J was investigated with the Cronbach’s alpha coefficient and test-retest reliability. In total, 112 of 130 respondents repeated the questionnaires after a 1-week interval; those answers were compared with the first round answers using intra-class correlation coefficients (ICC). The sample size for ICC analysis was determined based on the following assumptions. The null hypothesis (H0) was that the ICC would be 0.70, and the alternative hypothesis (H1) was that the ICC would be 0.90. With a power of 0.80, the minimum sample size required was 19 women. In the present study, our test-retest reliability sample (112 participants) satisfied the required sample size.

### Association between perceived injustice and severity of dysmenorrhea

We performed multivariable regression analysis to examine the association between perceived injustice and severity of menstrual pain. We calculated variance inflation factor (VIF) to examine multicollinearity in regression analysis. Hair et al. suggested that VIF < 10 was indicative of inconsequential collinearity [17]. The IEQ-chr-J was considered the independent variable, and we evaluated severity of dysmenorrhea by pain intensity and interference. We used log-transformed scores for the IEQ-chr-J, HADS anxiety, and HADS depression, as HADS anxiety and HADS depression scores were not normally distributed. We adjusted models for log (HADS anxiety), log (HADS depression), age, body mass index (kg/m^2^, categorized in quartiles), existence of irregular menstrual period (self-reported menstrual cycle from 25 to 35 days; yes or no), and endometriosis diagnosed by physician (yes or no). The 10 excluded women with hormone therapy might have included women with menstrual pain induced by endometriosis, because those with menstrual pain tend to take hormonal therapy; therefore, we re-ran models including these 10 women as a sensitivity analysis.

Respondents described their maximum/average menstrual pain intensity in the past 3 months using a NRS from 0 (no pain) to 10 (worst pain imaginable). An NRS is recommended when pain intensity is evaluated in clinical trials [18]. If respondents usually took analgesics for menstrual pain, they answered by assuming their pain intensity without taking analgesics. Pain interference was measured by the BPI pain interference subscale [19]. The BPI assesses pain interference in seven daily activities on a scale from 0 (does not interfere) to 10 (interferes completely): general activity, mood, walking, work, relationships with others, sleep, and enjoyment of life. Mean values of the scores for these seven activities were used to evaluate pain interference severity [20].

The significance level for the statistical hypothesis testing was set at *p* < 0.05. CFA was performed using IBM SPSS Amos version 25 (IBM Corp., New York, USA), and the sample size for ICC analysis was determined using PASS software version 13 (NCSS, Utah, USA). Other statistical analyses were performed using SAS version 9.4 (SAS Institute Inc., North Carolina, USA).

## Results

The mean values and proportions of participants’ characteristics are shown in Table 1. The mean maximum menstrual pain intensity was 6.5 (of 10 points), that of average menstrual pain intensity was 5.7, and that for impairment due to menstrual pain was 5.1.

**Table 1:** Means and proportion of characteristics (*n* = 130)

Age, year (SD)	33 (7.8)
Body mass index ≥25	16 (12.3)
Irregular menstruation, n (%)	15 (11.5)
Diagnosed as endometriosis, n (%)	6 (4.6)
Hormone drug usage, n (%)	10 (7.7)
Analgesic medicine usage, n (%)	72 (55.4)
IEQ-chr-J (SD)	14.2 (13.0) /48
PCS (SD)	20.9 (14.5) /52
HADS anxiety (SD)	7.7 (4.5) /21
HADS depression (SD)	7.3 (4.9) /21
Maximum menstrual pain intensity (SD)	6.5 (2.3) /10
Average menstrual pain intensity (SD)	5.7 (2.2) /10
Impairment due to menstrual pain (SD)	5.1 (2.5) /7

*SD* Standard deviation, *IEQ-chr-J* Japanese version of Injustice Experience Questionnaire-chronic, *PCS* Pain Catastrophizing Scale, *HADS* Hospital Anxiety and Depression Scale

**Fig. 1:**
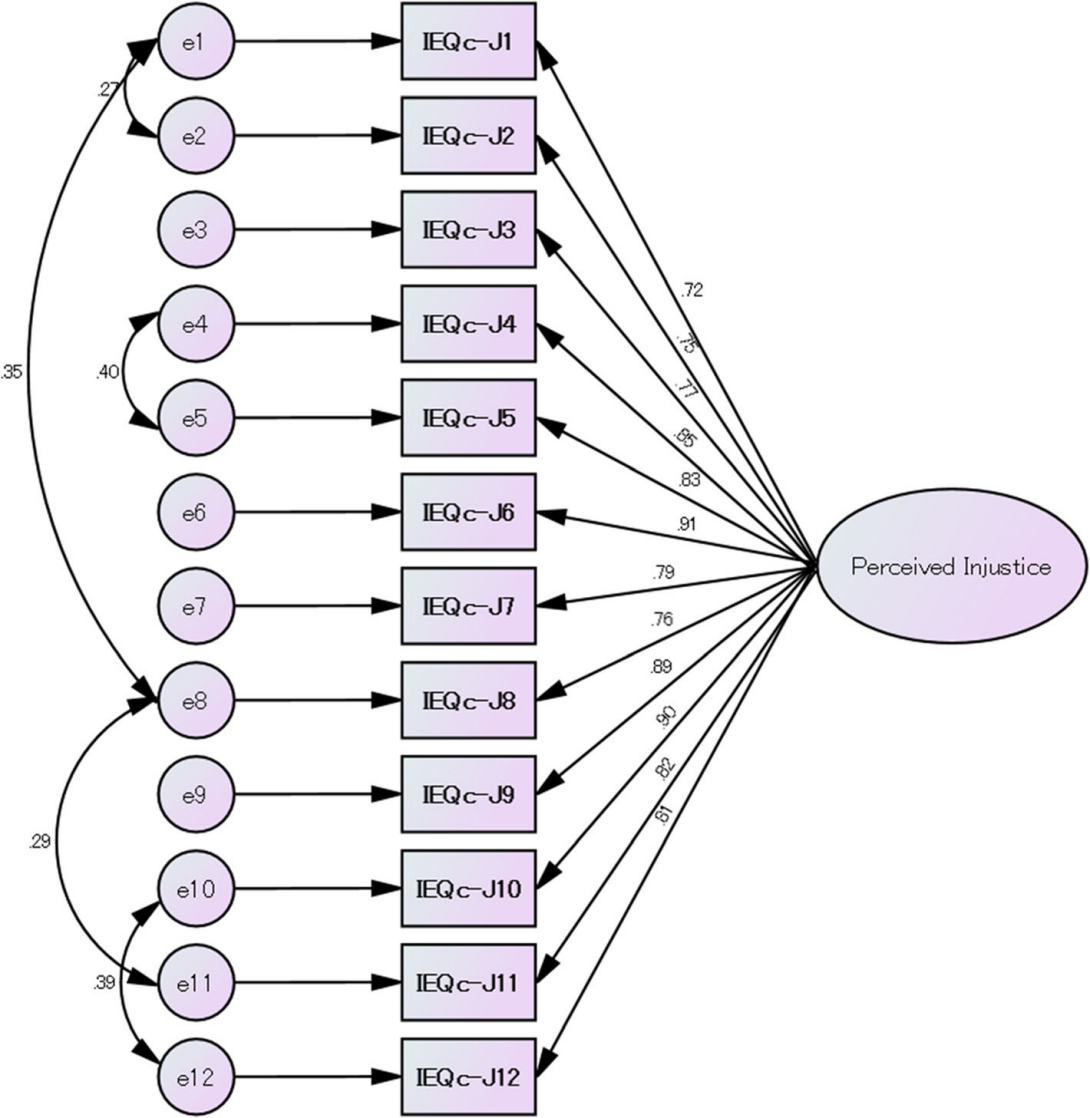
The one-factor model of the IEQ-chr-J with error terms e1–e12, and standardized parameter estimates

To evaluate validity, we determined five error covariance terms based on ≥9 points of the modification indices produced by the statistical software. Table 2 presents the goodness-of-fit summary for a one-factor model (Model 1) and a one-factor model including the five error covariance terms (Model 2). Although the SRMR (0.04) indicated a good fit, the RMSEA, CFI, and TLI showed a poor fit for Model 1, with values of 0.13 (90% CI: 0.11–0.16), 0.92, and 0.90, respectively. The fitness indices in Model 2 showed a good fit: RMSEA of 0.09 (90% CI: 0.07–0.12), SRMR of 0.03, CFI of 0.96, and TLI of 0.95. Figure 1 shows the one-factor model of the IEQ-chr-J with error terms e1–e12, and standardized parameter estimates ranging from 0.72–0.91.

**Table 2:** Goodness-of-fit summary (one-factor solution) (*n* = 130)

	χ^2^(df)	χ^2^/df	RMSEA (90% CI)	SRMR	CFI	TLI
Model 1: one factor (12 items)	179.20 (54)	3.32	0.13 (0.11–0.16)	0.04	0.92	0.90
Model 2: one factor (12 items + five error covariance)	103.32 (49)	2.11	0.09 (0.07–0.12)	0.03	0.96	0.95

χ2, chi square; df, degrees of freedom; RMSEA, root mean square error of approximation; CI, confidence intervals; SRMR, standardized root mean square residual; CFI, comparative fit indices; TLI, Tucker-Lewis Index

Table 3 shows the correlation coefficients with 95% CIs for the IEQ-chr-J, PCS, HADS anxiety, HADS depression, maximum and average menstrual pain intensity, and impairment due to menstrual pain in the past 3 months. Those correlation coefficients ranged from 0.27–0.65 (*p* < 0.05). The Cronbach’s alpha for the IEQ-chr-J was 0.96, and the ICC for test-rest reliability was 0.75 (95% CI: 0.61–0.88).

**Table 3:** Correlations coefficient (95%CI) (*n* = 130)

	PCS	HADS anxiety	HADS depression	Maximum menstrual pain intensity	Average menstrual pain intensity	Impairment due to menstrual pain
IEQ-chr-J	0.64 (0.53–0.73)^‡^	0.65 (0.54–0.74)^‡^	0.62 (0.50–0.71)^‡^	0.27 (0.11–0.43)^†^	0.27 (0.10–0.42)^†^	0.53 (0.39–0.64)^‡^

*IEQ-chr-J* Japanese version of Injustice Experience Questionnaire-chronic, *PCS* Pain Catastrophizing Scale, *HADS* Hospital Anxiety and Depression Scale. † *p* <0.01, ‡ *p* <0.001

Associations between respondents’ characteristics and severity of dysmenorrhea by multivariable regression analyses are shown in Table 4. Log (IEQ-chr-J) was only associated with impairment due to menstrual pain, with a standardized regression coefficient (β) of 0.36 (95% CI: 0.14–0.58). Log (IEQ-chr-J) was not correlated with maximum or average pain intensity. Although associations between diagnosed endometriosis and pain intensity/interference were not observed, the sensitivity analysis (including 10 women with hormonal therapy) indicated that diagnosed endometriosis was associated with average pain intensity, but not with maximum pain intensity or pain interference. The β (95% CI) in single regression analyses were 0.20 (0.02–0.37) for average pain intensity, 0.16 (− 0.01–0.33) for maximum pain, and 0.10 (− 0.07–0.28) for pain interference (data not shown in tables). VIF ranged from 1.03 to 2.39 in those regression analyses.

**Table 4:** Multivariable regression analysis examining the association between sample characteristics and severity of menstrual pain (*n* = 120)

		β (95%CI)	*R* ^2^	F
Dependent = maximum menstrual pain			0.11	0.38
	log IEQ-chr	0.22 (−0.03–0.48)		
	log HADSanxiety	0.14 (− 0.13–0.41)		
	log HADSdepression	−0.08 (− 0.36–0.19)		
	Age	−0.08 (− 0.27–0.11)		
	Body mass index	0.04 (−0.14–0.22)		
	Irregular menstruation	−0.08 (− 0.27–0.10)		
	Diagnosed as endometriosis	0.12 (−0.06–0.30)		
Dependent = average menstrual pain			0.11	0.33
	log IEQ-chr	0.22 (−0.03–0.48)		
	log HADSanxiety	0.19 (−0.08–0.46)		
	log HADSdepression	−0.14 (− 0.41–0.14)		
	Age	0.01 (−0.19–0.19)		
	Body mass index	0.03 (−0.16–0.21)		
	Irregular menstruation	−0.04 (− 0.22–0.14)		
	Diagnosed as endometriosis	0.13 (−0.05–0.31)		
Dependent = impairment due to menstrual pain			0.35	0.22
	log IEQ-chr	0.36 (0.14–0.58)‡		
	log HADSanxiety	0.22 (−0.1–0.46)		
	log HADSdepression	0.06 (−0.18–0.29)		
	Age	−0.04 (− 0.21–0.12)		
	Body mass index	−0.06 (− 0.22–0.09)		
	Irregular menstruation	−0.01 (− 0.16–0.15)		
	Diagnosed as endometriosis	−0.05 (− 0.20–0.10)		

*β* standerdized regression coefficient, *IEQ* Injustice Experiences Questionnaire

*HADS* Hospital Anxiety and Depression Scale

‡ *p* <0.001

## Discussion

The present study found that the IEQ-chr-J had acceptable psychometric properties with evident validity and reliability, and found a strong correlation between perceived injustice and impairment due to menstrual pain. To our knowledge, this is the first study reporting an association between perceived injustice and menstrual pain.

Experimental research suggests that injustice appraisals are likely to trigger anger responses [21–23]. A recent investigation conducted with patients with long-standing chronic pain demonstrated that anger mediated the relation between scores on a measure of perceived injustice and pain severity [24]. There are indications that anger might exacerbate the intensity of pain by increasing muscle tension and by inhibiting endogenous opioid analgesia [25, 26]. Anger may thus be one vehicle through which perceived injustice contributes to symptom severity.

It has been suggested that justice-related appraisals might lead individuals to ruminate or focus excessively on their suffering or losses, ultimately increasing their physical and emotional distress. Attributional processes have also been discussed in relation to perceptions of injustice [21]. To the degree that individuals with high levels of perceived injustice make external attributions (i.e., blame others) for their negative circumstances, they may be less likely to take responsibility for mitigating their losses. External attributions might promote a more passive orientation to managing one’s health condition, in turn contributing to more marked life impairment.

On the basis of past research findings, we hypothesized that perceived injustice would be associated with higher intensity of menstrual pain and impairment. Zero order correlations revealed significant associations between perceived injustice and menstrual pain and impairment. In regression analyses, the relation between perceived injustice and menstrual impairment remained significant even when controlling for confounders, notably anxiety and depression. However, perceived injustice did not emerge as a unique predictor of the severity of menstrual pain when confounders were statistically controlled. This pattern of findings is consistent with what has been reported in the literature. Sullivan et al. have proposed that perceived injustice might be more strongly related to disability than pain severity [3].

Previous study reported it is often difficult for injured individuals with severe perceived injustice to return to work [3] and that effective behavioral activation interventions targeting perceived injustice are necessary [7–9]. Intervention techniques may need to be designed to reduce perceived injustice, including validation, empathic reflection, guided disclosure, thought monitoring, problem solving, and goal setting [7–9]. Perceived injustice may also be a therapeutic target among patients with severe primary dysmenorrhea.

Although endometriosis is known to be a major cause of menstrual pain, diagnosed endometriosis was not associated with menstrual pain in this study. This was possibly because of the small power (endometriosis *n* = 6) or because respondents with severe menstrual pain due to endometriosis had already been treated by hormone drugs and were therefore excluded when we analyzed these data.

The present study had some limitations. First, our study used a cross-sectional design, and therefore we cannot discuss temporal aspects. Second, we used data of web-based survey, which includes potential disadvantages compared with face-to-face interviews and surveys using a paper questionnaire; we cannot negate the possibility that our participants might not be a representative sample [27]. For example, only individuals who could sign up for the website would be able to participate in online surveys. This possibility of a non-representative sample could have influenced the results. Third, our respondents were not menstruating at the time they answered the questionnaire and had to recall their menstrual pain over the past 3 months. This created the possibility of recall bias.

## Conclusions

We found the IEQ-chr-J to have acceptable psychometric properties to measure perceived injustice. We also found that perceived injustice is associated with impairment due to menstrual pain, suggesting that perceived injustice among women with primary dysmenorrhea may be a risk factor for impairment due to menstrual pain.

## Additional file

Additional file 1: Items of IEQ and IEQ-chr. (DOCX 14 kb)

## Data Availability

Data cannot be shared publicly because datasets have ethical or legal restrictions for public deposition owing to inclusion of sensitive information from the human participants. Based on regulations for ethical guidelines in Japan, the Institutional Review Board for Clinical Research of Juntendo University Hospital imposed restrictions on the data collected in this study. All enquiries should be addressed to the Data Management Committee via email: kenkyu5858@juntendo.ac.jp

## Abbreviations

β: a standardized regression coefficient
BPI: the Brief Pain Inventory
CFA: confirmatory factor analysis
CFI: comparative fit index
CI: confidence intervals
HADS: the Hospital Anxiety and Depression Scale
ICC: intra-class correlation coefficients
IEQ-chr-J: a Japanese version of the Injustice Experience Questionnaire-chronic
NRS: a numerical rating scale
PCS: the Pain Catastrophizing Scale
RMSEA: the root mean square error of approximation
SRMR: the standardized root mean square residual
TLI: Tucker-Lewis index
VIF: variance inflation factor
χ^2^ /df: chi square divided by degree of freedom
χ^2^: chi square
