# Supplementary material for: Developing a Japanese version of the Injustice Experience Questionnaire-chronic and the contribution of perceived injustice to severity of menstrual pain: a web-based cross-sectional study
Source: Biopsychosoc Med. 2019 Jul 22;13:17. doi: 10.1186/s13030-019-0158-z (PMC6643310; doi:10.1186/s13030-019-0158-z)
Supplement: Supplementary file 1 — Items of IEQ and IEQ-chr. (DOCX 14 kb) [file 13030_2019_158_MOESM1_ESM.docx]

**S1. Appendix**

Both IEQ and IEQ-chr use a 5-point Likert-type scale, ranging from 0 (never) to 4 (all the time), and the items of IEQ and IEQ-chr are as follows:

*Item 1, “Most people don’t understand how severe my condition is”, item 2, “My life will never be the same”, item 3, “I am suffering because of someone else’s negligence”, item 4, “No one should have to live this way”, item 5, “I just want to have my life back”, item 6, “I feel that this has affected me in a permanent way”, item 7, “It all seems so unfair”, item 8, “I worry that my condition is not being taken seriously”, item 9, “Nothing will ever make up for all that I have gone through”, item 10, “I feel as if I have been robbed of something very precious”, item 11, “I am troubled by fears that I may never achieve my dreams”, and item 12, “I can’t believe this has happened to me”.*

(Cited from, Sullivan MJL, Adams H, Horan S, Maher D, Boland D, Gross R. The role of perceived injustice in the experience of chronic pain and disability: Scale development and validation. J Occup Rehabil. 2008;18(3):249–61.)
